# Supplementary material for: Brain health index as a predictor of possible vascular dementia in the Mexican health and aging study 2012–2015
Source: PLoS One. 2024 May 23;19(5):e0304234. doi: 10.1371/journal.pone.0304234 (PMC11115212; doi:10.1371/journal.pone.0304234)
Supplement: S1 Table — (DOCX) [file pone.0304234.s001.docx]

**Supplementary Table 1.** Demographic characteristics of MHAS sample 2012 by diagnostic group

| Characteristics | Total  n=14,890 | Cognitively unimpaired  n=13,899 | Dementia  n=653 | Stroke    n=254 | Possible Vascular Dementia  n=84 | p-value |
| --- | --- | --- | --- | --- | --- | --- |
| Age* Mean (SD) | 63.0 (9.8) | 62.5 (9.4) | 72.8 (11.6) | 63.9 (10.9) | 78.1 (9.6) | <.001 |
| Female (%) | 53.3 | 53.2 | 59.4 | 41.7 | 55.1 | <.001 |
| Education (y)* Mean (SD) | 5.7 (4.8) | 5.8 (4.8) | 3.4 (3.5) | 4.7 (4.0) | 4.0 (4.8) | <.001 |
| Hypertension (%) | 37.8 | 36.7 | 52 | 64.8 | 63.2 | <.001 |
| Diabetes (%) | 19.5 | 18.8 | 30.6 | 34.2 | 17.4 | <.001 |
| Myocardial infarction (%) | 3.1 | 2.6 | 8.0 | 13.1 | 26.8 | <.001 |
| Obesity (%) | 24.6 | 25.0 | 11.8 | 30.4 | 18.9 | <.001 |
| Smoking (%) | 39 | 39.2 | 30.4 | 48 | 31.4 | <.001 |
| Physical inactivity (%) | 59.2 | 58.4 | 84.3 | 77.5 | 48.3 | <.001 |
| Depressive symptoms (%) | 31.8 | 30.7 | 63.1 | 52.5 | 61 | <.001 |
| BHI score*  Mean (SD) | 2.0 (1.2) | 1.9 (1.2) | 2.9 (1.2) | 3.0 (1.2) | 2.6 (0.9) | <.001 |

y: years, SD: Standard deviation, BHI: Brain Health Index. P-value from ANOVA for continuous variables and Chi-square for categorical variables. All values were weighted and derived from the MHAS sampling weights. Bonferroni correction; *All comparisons between groups were significant (p<.001), **Comparisons were significant (p<.001), except for possible vascular dementia *vs* cognitively unimpaired.
