# Supplementary material for: Brain health index as a predictor of possible vascular dementia in the Mexican health and aging study 2012–2015
Source: PLoS One. 2024 May 23;19(5):e0304234. doi: 10.1371/journal.pone.0304234 (PMC11115212; doi:10.1371/journal.pone.0304234)
Supplement: Attachment — Submitted filename: Response to reviewers.docx [file pone.0304234.s002.docx]

January 12, 2024

Response to reviewers
PLOS ONE Journal

Dear editor in chief, academic editor, and reviewer:

We are thankful for the thoughtful comments on our manuscript. Your valuable and insightful comments have led to possible improvements in the current version. The authors have carefully considered the comments and tried our best to address each one of them. Moreover, the authors welcome further constructive comments if any.

In this letter, our responses are written in purple. We have included a marked-up copy of the manuscript where changes are highlighted in yellow.

**In response to Journal’s additional requirements**:

1. **Please ensure that your manuscript meets PLOS ONE's style requirements, including those for file naming.**

We have modified the manuscript’s, title (lines 2-3) and abstract section (lines 28-49) to meet PLOS One’s manuscript title, author, affiliations, and body formatting guidelines.

2. **Funding Information and Financial Disclosure sections do not match**.

We have corrected the mentioned sections and included a statement in the revised cover letter; “The authors declare that they have no conflicts of interest and that this research has not received any financial funding.”

3. **Data availability statement**.

We would like to change our availability statement as follows: “Data are available from The Mexican Health and Aging Study (MHAS) from [www.mhasweb.org](http://www.mhasweb.org)”.

The manuscript’s methodology section (lines 118-119) includes an e-link to access the MHAS data and documentation, which are of public use and available at www.mhasweb.org. If specific data is required, please let us know.

4. **Please review your reference list to ensure that it is complete and correct**.

Thank you for your suggestion. We have carefully reviewed the reference list and corrected a mistake in line 295, where an author’s first name was misplaced. We have ensured this list is complete and correct. We did not include any retracted papers.

**In response to Reviewer #1:**

1. **The abstract objective and the main text must be consistent. Please clarify.**

We are very thankful of your comment. We have homogenized the concepts included in the abstract (lines 33-35) for it to be consistent with the objective described in the main text (lines 101-102).

2. **Specify the study design in the methods section**.

We are grateful you have identified this. We have addressed this issue in line 105, 109-112.

3. **In the discussion section, it would be helpful to claim why the BHI was adapted for the Mexican population as the proposal in this paper**.

We are very thankful of your suggestion. We have added a sentence as to why it is important to adapt the BHI to the Mexican population in lines 254-257.

4. **Suggestions in lines 59, 60, 64, and 119**.

Thank you for your suggestions. In this revised version of the manuscript, we have addressed these issues in lines 52, 54, 57, and 113, respectively.

5. **Why is the INCMNSZ in Spanish and other institutions, as INEGI is in English**.

Thank you for identifying this. We have included this Institution’s name (INCMNSZ) in English (line 114).

6. **To help the reader, it is better if the description of the table in lines 241 - 246 is in the same order as the table**.

We are very thankful of your comment. We have updated Table’s 2 description in lines 234-238.

7. **In Table 2, please use a * in the Sex word to help the reader see that the male sex was used for the multinominal regression model. The word multinominal is misspelled**.

We appreciate your comment and have corrected this issue in Table 2 (lines 241-245).

Thank you for your consideration.

Sincerely,

Sara Gloria Aguilar Navarro, PhD
sara.aguilarn@incmnsz.mx
Professor, Deparment of Geriatric Medicine,
Instituto Nacional de Ciencias Médicas y Nutrición Salvador Zubirán, Tlalpan, Mexico City, Mexico
